# Chikungunya, Dengue, and Malaria Co-Infection after Travel to Nigeria, India 

**DOI:** 10.3201/eid2105.141804

**Published:** 2015-05

**Authors:** C.G. Raut, N.M. Rao, D.P. Sinha, H. Hanumaiah, M.J. Manjunatha

**Affiliations:** National Institute of Virology–Bangalore Unit, Bengaluru, India

**Keywords:** abrbovirus, chikungunya, dengue, malaria, parasites, vector-borne infections, mosquitoes, zoonoses, Nigeria, India, travel, viruses

**To the Editor:** Arboviral infections, such as chikungunya and dengue, are endemic to South Asia. Concurrent infection of these viral infections with another vector-borne parasitic disease, malaria, is uncommon in India and would pose a challenge for medical diagnosis because of overlapping clinical symptoms. We present a case of multiple co-infections in a young man attending college in India after his return from Nigeria, a region endemic for chikungunya, dengue, and malaria.

After spending a 1-month vacation in Nigeria, a 21-year-old male asymptomatic Nigerian national arrived in Bengaluru, India, on September 5, 2014, to resume college. He developed febrile illness, chills, abdominal discomfort, headache, epigastric pain, and myalgias 6 days after his arrival. High-grade fever (103°F), icterus, and vomiting subsequently developed. He received treatment for his symptoms, and a physical examination revealed general weakness, pulse rate of 100 beats/min, and blood pressure of 140/70 mm Hg.

Various tests to assess his medical condition were conducted. A complete blood count showed a reduced platelet count of 68,000/mm^3^ (reference 1.5–5.0 mm^3^); findings of an abdominal ultrasonography were normal. Comprehensive kidney and liver function tests showed elevated values (blood urea, 53 mg/dL [reference 15–45 mg/dL]; serum creatinine, 1.66 mg/dL [reference 0.6–1.2 mg/dL]; aspartate aminotransferase, 67 IU/L [reference 5–34 IU/L]). Because the man had visited and returned from a region endemic for chikungunya, dengue, and malaria (*1*) and had a reduced platelet count, diagnostic tests for these infections were conducted. Accordingly, dengue nonstructural 1 antigen detection rapid test conducted on blood collected 2 days after symptom onset was positive. Microscopic observation of thick and thin blood smears (also from blood taken 2 days after symptom onset) showed the malaria parasite *Plasmodium falciparum*. Reverse transcription PCR (RT-PCR) on serum collected 2 days after symptom onset was conducted to detect chikungunya and dengue viral genomes. Test results were positive for both chikungunya and dengue viruses. However, IgM antibody capture–ELISA (MAC-ELISA) for detecting chikungunya and dengue viral antibodies was negative for both infections. In accordance with World Health Organization travel guidelines, a blood sample, taken 3 days after symptoms onset, was tested at the National Institute of Virology (Pune, India) for Ebola virus disease. This test used RT-PCR and real-time RT-PCR to detect Ebola virus nucleoprotein and polymerase genes and ruled out Ebola virus disease.

These tests were repeated with standard positive and negative controls to ensure no contamination and no false-positive results. RT-PCR for chikungunya and dengue viruses was performed by using virus gene–specific primers. RT-PCR for Japanese encephalitis and West Nile viruses also were conducted to rule out these cross-reacting arboviral infections that share common clinical manifestations with chikungunya and dengue. The failure of MAC-ELISA to detect chikungunya virus– and dengue virus–specific IgM was attributed to collection of the blood on day 2 after symptom onset, and thus the IgM would not have been generated to be detected by MAC-ELISA.

Fever similar to those common with malaria and typhoid are often exhibited with any of the arboviral infections that are endemic to Nigeria (*1*). Often these fevers are misdiagnosed as malarial fevers, and the opportunity to test for arboviral infections is missed. Dual infections of chikungunya and dengue are becoming more common in India (*2*,*3*), and there were earlier reports of dengue and malaria co-infection (*4*). Because these diseases are endemic to both Nigeria and India and because the incubation periods of infections vary, we do not know the exact location where the patient acquired any or all of these infections. Multiple infections in a single patient would drastically change the spectrum of clinical manifestations and thus complicate the diagnosis process. Our study particularly draws attention to understanding emerging arboviral infections and emphasizes the need for a multidimensional diagnostic approach in such clinical situations.
